# Using the Staircase Approach to increase movement: a systematic search and review to inform a novel sedentary behaviour intervention for older adults

**DOI:** 10.24095/hpcdp.45.2.01

**Published:** 2025-02

**Authors:** Konstantina Katsoulis, Maria C. Tan, Sean Horton, Samir K. Sinha, Bill Kapralos, David Dunstan, Danielle R. Bouchard, Jennifer L. Copeland, Shilpa Dogra

**Affiliations:** 1 Ontario Tech University, Oshawa, Ontario, Canada; 2 University of Windsor, Windsor, Ontario, Canada; 3 Division of Geriatric Medicine, Department of Medicine, University of Toronto, Toronto, Ontario, Canada; 4 Section of Geriatric Medicine, Department of Medicine, Sinai Health and University Health Network, Toronto, Ontario, Canada; 5 National Institute on Ageing, Toronto Metropolitan University, Toronto, Ontario, Canada; 6 Institute for Physical Activity and Nutrition, Deakin University, Melbourne, Victoria, Australia; 7 Baker Heart and Diabetes Institute, Melbourne, Victoria, Australia; 8 Cardiometabolic, Exercise & Lifestyle Laboratory, University of New Brunswick, Fredericton, New Brunswick, Canada; 9 University of Lethbridge, Lethbridge, Alberta, Canada

**Keywords:** sedentary time, sitting, physical inactivity, behaviour change, intervention strategies, intervention design

## Abstract

**Introduction:**

Traditional approaches to supporting older adults in adopting and maintaining an active lifestyle have largely failed. The previously proposed “Staircase Approach” offers a new foundation for developing interventions and public health strategies; this approach includes Step 1 (changing sedentary behaviour) and Steps 2 to 4 (incorporating more physical activity of increasing levels of intensity). In this systematic search and review, we aimed to inform the co-creation of a novel Staircase Approach intervention for community-dwelling, inactive older adults, primarily focussed on Step 1.

**Methods:**

A systematic search was performed across six databases (MEDLINE, PsycInfo, CINAHL, Cochrane CENTRAL, SPORTDiscus and Scopus).

**Results:**

After duplicates were removed, 3427 titles and abstracts were screened. Fourteen articles (including 17 intervention groups) were included after full-text review. Five were randomized controlled trials, three compared two interventions and six were single-arm studies. Sample sizes ranged from 9 to 176 participants, and included 617 older adults at baseline. Mean age of samples ranged from 64.3 (standard deviation [SD] 3.8) to 85.1(SD 6.2) years, while the intervention length ranged from less thanone day to 6 months. Sedentary time interventions are well accepted; most studies had completion rates above 80%. Based on findings from within-group comparisons, half of the studies showed a reduction in sedentary time (6/12 groups) and half showed an increase in physical activity (6/12 groups). Based on findings from between-group comparisons, 2 out of 5 intervention groups showed improvements in sitting time and physical activity outcomes compared to controls. Satisfaction and adherence to interventions were generally high.

**Conclusion:**

Sedentary time interventions for older adults show promise and point to several components that may be included in an intervention focussed on Step 1 of the Staircase Approach.

**Highlights**

- Sedentary behaviour interventions for community-dwelling older adults have been positively received, and demonstrate good adherence, acceptability and completion rates.
- Past research suggests that it may be necessary to consider several intervention components when cocreating a sedentary behaviour reduction intervention specifically for older adults.
- Researchers must be more consistent in their reporting of behaviour change techniques incorporated into interventions to provide insights into the development of new interventions.

## Introduction

Older adults (aged ≥65 years) have the lowest reported rates of physical activity and accumulate the highest volume of sedentary time.^1^,^2^ Global device-based data indicate that older adults are accumulating an average of nine hours of sedentary time (during waking hours) per day.^3^ This high volume of sedentary time is associated with declines in important outcomes such as physical function, cognitive function, mental health, sleep quality and social engagement.^4^,^5^

Research suggests that high volumes of moderate-intensity physical activity (60–75 min/day) are needed to overcome the detrimental effects of sedentary time.^6^ Unfortunately, only 5% to 20% of older adults are meeting minimum recommendations of physical activity (150 min/week);^1^ worse still, these recommendations fall far below the volume needed to negate the effects of sedentary time. Over the past few decades, physical inactivity levels have tended to remain stable,^7^ indicating that efforts to increase physical activity among older adults have been largely unsuccessful. Thus, new approaches are urgently needed to support the growing population of older adults with integrating movement into their lives. 

We have previously proposed that the lack of success in increasing physical activity participation may be attributable to the magnitude of the initial goal being set.^4^ Specifically, it may be prudent to start with smaller, more achievable goals related to movement that can activate individuals along a pathway to an active lifestyle using a progressive, stepwise approach. Our previously proposed “Staircase Approach” begins with focussing on reducing sedentary time by encouraging less total sitting and more interrupted sitting (e.g. increased sit-to-stand transitions; Step 1) before progressing to physical activity that ranges from light-intensity (Step 2) to moderate and vigorous intensities (Steps 3–4).^4^

This approach is distinct from previous strategies that have either focussed on these movement behaviours separately, or on both simultaneously, rather than using a sequential or stepwise approach. Older adults with multiple comorbidities or functional limitations may not be able to start with physical activity. Thus, starting with sedentary time–related goals may be more prudent. 

Spending less time in sedentary behaviours may lead to health benefits. For example, a study of older adults (M 73.3, standard deviation [SD]5.9, years) found that breaks in sedentary time were significantly associated with the arm curl, the chair stand test and composite physical function scores, even after adjusting for moderate-vigorous physical activity and total sedentary time.^8^ Such findings support the importance of stressing that sitting less (Step 1) should be considered a success even if physical activity is never increased to the recommended levels. Furthermore, it has been estimated that significant economic benefits are associated with achieving this initial goal of reducing sedentary time. Specifically, it has been estimated that in Canada, a 10% decrease in excessive sedentary behaviour (from 87.7%–77.7%) would save an estimated CAD 219 million per year in related costs.^9^

The traditional approach of focussing on physical activity has led to the development of a plethora of community-based programs available to older adults.^10^-^12^ However, there is a significant gap in programming available to support community-dwelling older adults who need interventions focussed on Step 1 before potentially progressing to Steps 2 through 4. To inform the design of an intervention for community-dwelling older adults focussed on starting with Step 1, we conducted a systematic search and review of the literature.^13^ This type of review “combines strengths of critical review with a comprehensive search process” and results in a “best evidence synthesis.”^13^^,p.95^

Previous reviews on interventions designed to reduce sedentary time in older adults have excluded older adults with common health conditions (e.g. stroke),^14^ focussed primarily on cardiometabolic health markers^15^ or included active older adults at baseline.^16^ Our approach was focussed on community-dwelling older adults who are inactive and sedentary; the intent was to inform an intervention that could be used in this broad population of older adults. Thus, the aim of this review was to synthesize knowledge from interventions designed to decrease sedentary time in inactive, community-dwelling older adults (regardless of health status and functional autonomy) to inform the development of a progressive behaviour-change intervention.

## Methods

The study protocol was registered in Open Science Framework.^17^ We used the PRISMA checklist to ensure proper reporting (available from the authors upon request).^18^

***Eligibility criteria***

Studies were considered eligible for the current review if they fulfilled the following inclusion criteria: (1) participants were aged 60 years or older, (2) participants were living in the community, (3) participants were described as sedentary and/or inactive at baseline by study authors or as part of the eligibility criteria using either self-report or device-measured movement, (4) the study intervention was a behaviour change intervention and (5) the intervention took place in a community-based setting. 

Studies were excluded if they were (1) laboratory- or gym-based, supervised exercise programs, (2) qualitative-only studies, (3) only designed to assess health-related outcomes and did not measure sedentary time/behaviour, (4) protocols, (5) editorials or opinion pieces, (6) conference abstracts, (7) dissertations or (8) written in a language in which no one from the team was fluent enough to review (i.e. languages other than English, Hindi and Greek). Nonrandomized intervention studies were included as long as they met all other inclusion and exclusion criteria.

***Information sources***

A systematic search of the following bibliographic databases was conducted by a health sciences librarian: (1) Ovid MEDLINE (1946 to 13 June 2023); (2) EBSCOhost CINAHL Plus with Full Text (1937 TO 14June 2023); (3) EBSCOhost SPORTDiscus (database inception to 14 June 2023); (4) ProQuest APA PsycInfo (inception to 14 June 2023); (5) EBM Reviews - Cochrane Central Register of Controlled Trials (inception to May 2023); and (6) Scopus (inception to June 2023).

***Search strategy***

Searches were conducted on 14 June 2023. The librarian (MCT) developed the MEDLINE search strategy in consultation with the team. The MEDLINE strategy was peer reviewed by another expert searcher via the Peer Review of Electronic Search Strategies (PRESS) forum, revised per feedback and adapted for each included bibliographic database. The search strategies combined relevant subject headings (e.g. MeSH) and keywords relevant to the concepts of older adults and sedentary time reduction. No language, date or study design limits were applied. Database search results were imported to Covidence^19^ for deduplication and screening. 

***Selection process***

Titles and abstracts were screened in dual reviewer mode in Covidence. All references were reviewed once by a single researcher (KK), with the second vote performed by other members of the research team (n=3). Ties were decided by SD. For full-text screening, two researchers (SD and KK) screened articles for eligibility, and any ties were settled with discussion. 

***Data collection process***

Data were manually extracted from each study by KK based on the items identified below. A second researcher (SD) checked all data in the extraction table. Data for development of our tables were then further extracted and verified by KK. 

***Data items***

The following information was extracted from the studies: (1) study identification number, (2) title, (3) study aim, (4) country, (5) study design, (6) control/comparison group, (7) sample characteristics/demographics, (8) inclusion criteria, (9)exclusion criteria, (10) method of recruitment, (11) intervention description, (12) types of behaviour change techniques used, (13)main outcomes of interest, (14) other outcomes, (15) sample size of group(s) at baseline and postintervention, (16) dropouts with reasons, (17) adverse events/injuries reported, (18) percentage completers, (19) engagement, (20) compliance/adherence, (21) main findings, (22) overall conclusions, (23) limitations, (24) strengths and (25) insights from the discussion. 

Data for primary outcomes—changes in movement behaviours, adherence and compliance—were identified and extracted. Changes in sitting time were reported either as total sedentary time (min/day), breaks in sedentary time (number/day), sit-to-stand transitions (number/day), or bouts of sedentary time (number/day or min/day). Changes in physical activity were reported as either changes in steps (count/day), or walking, light-intensity physical activity, moderate-intensity physical activity or vigorous-intensity physical activity (min/day). When movement behaviours were not measured using devices, self-reported data were extracted. The percentage of participants completing the intervention (“completers”) was calculated as: n at baseline/n at post-study 100. To understand which components of interventions are critical, data on behaviour change theories, behaviour change techniques and intervention characteristics used in the studies were also extracted.

***Study risk of bias assessment***

Using the checklist for randomized controlled trials (RCTs) provided by the Scottish Intercollegiate Guidelines Network, the overall quality of a study can be classified as high, acceptable, low or unacceptable.^20^ Quality assessment is based on 10 items, with the emphasis on randomization and randomization methods. Given that this review focussed on behaviour change interventions and engagement-related outcomes, aspects of randomization such as concealment and blinding were not applicable, and in some cases, tool validity and reliability were not applicable. Thus, the risk of bias assessment was based on study design, such that RCTs were considered higher quality and non-RCTs were considered lower quality. For our classification purposes, RCTs did not include randomized studies in which a comparison to another sedentary behaviour intervention was made.

## Results

***Study selection***

**Figure 1:**
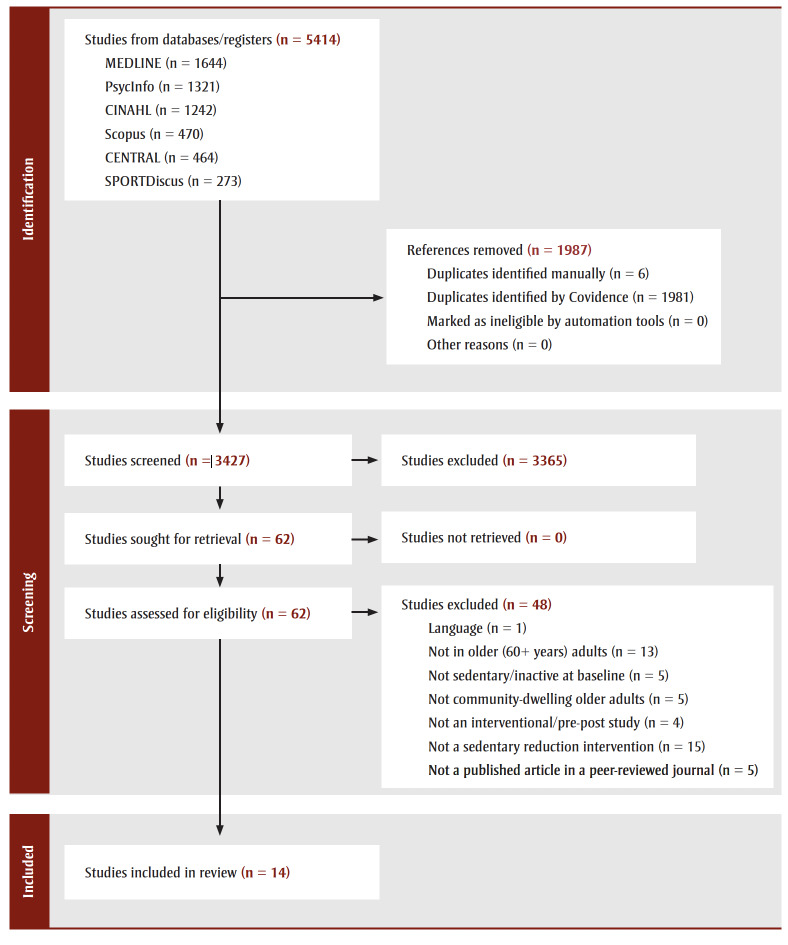
PRISMA diagram of search process

The search process yielded 5414 references from bibliographic databases, with 3427 references remaining once duplicates were removed ( Figure 1). Reasons for exclusion at the title/abstract level were a lack of mention of sedentary time/behaviour as an outcome of interest, a clear focus on exercise interventions, a nonintervention design and samples younger than 60 years. No additional references were identified from other sources. After full-text review, 14 articles were included in the final tally of the current review. Of the final included articles, 17 intervention groups were available, since three articles reported data for two intervention groups.^21^-^23^ For Tosi et al., the “control” group was included as an intervention group for the current review, since their participants were administered a sedentary behaviour intervention in the form of education.^23^

***Study characteristics***

The characteristics of each study can be found in Table 1. Of the 14 studies, five were RCTs,^21^,^24^-^27^ three reported on the results of two interventions via two arms,^22^,^23^,^28^ and six were single-arm, pre-post intervention studies with no control group.^29^-^34^ The sample sizes of intervention groups ranged from 9 to 176 participants and included a total of 617 older adults at baseline across all studies. The mean age of the participants ranged from 64.3(SD 3.8) to 85.1(SD 6.2) years across studies. The primary objective of six of the studies was to assess feasibility.^21^,^24^,^29^,^31^-^33^

**Table 1:** Characteristics and conclusions of the studies included

Study;	Participant	Study design	Length of	Primary movement	Movement	Conclusion
Aunger 2020; Dudley, UK	n=24 (14F, 10M); age: 73.3(SD 5.6) y; pre-surgery population	RCT; 2 groups	8 weeks	Reduce ST via personalized consultations	ST (device-based and self-report)	Study was feasible with some modifications.
Blair 2021 (A + B); Albuquerque, US	A: Tech support group: n=18 (8F, 10M); age: 69.6(SD4.5) y; B: Tech support + health coaching group: n=18 (12F, 6M); age: 69.1(SD 4.0) y; cancer survivors	RCT; 3 groups (2 interventions and controls)	13 weeks	Reduce ST via standing (prompt progressed from every 60 min to 30 min of prolonged sitting) and moving (steps progressed from 1000 to 3000 steps/day above baseline)	ST and PA (device-based)	Intervention was feasible and acceptable; there was no reduction in sedentary time or increase in breaks.
Burke 2013; Perth, Australia	n=176 (83F, 93M); age: 65.8(SD3.0) y	RCT; 2 groups (intervention vs. controls)	6 months	Personalized goals to reduce ST/increase PA	ST and PA (self-report)	Intervention was feasible and led to improvements in some PA outcomes.
Kleinke 2021; Greifswald, Germany	n=85 (50F, 35M); age: 70.4(SD4.6) y	RCT; 2 groups	6 months	Reduce ST via feedback letters based on activity monitors	ST and PA (device-based and self-report)	Intervention was not effective at changing PA levels in an active sample.
Rosenberg 2020; Seattle, US	Intervention group: n=29 (20F, 9M); age: 69(SD4.7) y; BMI: 35.7(SD5.9) kg/m2; Controls: n=31 (21F, 10M); age: 67.8 (SD 5.2) y; BMI=35.1 (SD3.7) kg/m2	RCT; 2 groups (intervention vs. controls)	12 weeks	Reduce ST via personalized goals of interrupting sitting and standing/moving, aiming for reduction of sitting of 60 min/day.	ST and PA (device-based)	Sitting time was reduced by increasing standing time.
Barone Gibbs 2017; Pittsburgh, US	n=19 (14F, 5M); age: 68.5(SD6.7) y	Randomized trial comparing 2 interventions	12 weeks	Reduce ST by 1 h/day via personalized consultations	ST and PA (device-based and self-report)	Intervention can be effective to promote increases in PA; targeting SB can have unique short-term benefits (functional performance).
Compernolle 2020; Ghent, Belgium	n=28 (15F, 13M); age: 64.3(SD3.8) y	Single-arm study	3 weeks	Reduce ST through self-monitoring and prompts to stand after 30 min of sitting	ST and PA (device-based)	Intervention was well received but no reduction in sitting time was found.
Fitzsimons 2013; Glasgow, UK	n=24 (10F, 14M); age 68(SD6) y	Single-arm study	2 weeks	Reduce ST via personalized consultations	ST and PA (device-based and self-report)	Intervention reduced ST.
Gardiner 2011; Queensland, Australia	n=59 (44F, 15M); age: 74.3(SD9.3) y	Single-arm study	One 45-min session	Reduce ST via personalized consultations; “Stand and move after 30 min of sitting”	ST and PA (device-based)	Brief behaviour change intervention can reduce ST.
Koltyn 2019 (A + B); Madison (WI), US	A: Study 1: n=12 (10F, 2M); age: 68.86(SD4.53) y; B: Study 2: n=9 (7W, 2M); age: 67.8(SD7.7) y	2 single-arm studies	4 weeks (Study 1) and 8 weeks (Study 2)	Study 1: stand 3–5 times/day up to 10–12 times/day Study 2: Similar to Study 1 with a refresher workshop at 6 weeks	ST and PA (device-based and self-report)	There were moderate effects for reducing ST and improving PA.
Lewis 2016; Adelaide, Australia	n=27 (17F, 10M); age: 71.7(SD6.5) y	Single-arm study	6 weeks	Reduce ST via personalized consultations; stand 15 min/day the first week and progress to 90 min/day by 6 weeks via 6 steps	ST and PA (device-based and self-report)	Intervention was feasible and led to reduction in sitting time in older adults.
Matei 2015; London, UK	n=23 (16F, 7M); age: 66.9(SD4.2) y	Single-arm study	8 weeks	Reduce ST by standing during commercials and after every 20 min of computer use, and increase general PA	SB and PA (self-report)	Intervention was generally acceptable and showed low attrition and moderate adherence among sedentary and inactive older adults.
Rosenberg 2015; Seattle, US	n=23 (16F, 7M); age: 71.4(SD6.4) y; BMI: 34, range: 27–47	Single-arm study	8 weeks	Reduce ST via personalized goals; increase standing and moving by 2 h/day and increase sit-to-stand transitions by 15/day	ST and PA (device-based and self-report)	Intervention was feasible and effective at reducing ST.
Tosi 2021 (A + B)^a^; Sao Paulo, Brazil	A: Intervention: n=21 (18F, 3M); age: 82.9(SD6.8) y; B: SB education controls: n=22 (19F, 3M); age: 85.1(SD6.2) y; majority had several chronic conditions and frailty	2 single-arm studies [randomized trial: 2 groups (intervention vs. “controls” that received SB education)]	16 weeks	A: reduce ST via personalized standing exercises (up to 30 min/day; B: general information on health consequences of SB	ST (device-based)	Intervention reduced SB and showed satisfactory adherence.

**Abbreviations:** BMI, body mass index; F, females; h, hour(s); M, males; min, minutes; PA, physical activity; RCT, randomized controlled trial; SB, sedentary behaviour; SD, standard deviation; ST, sedentary/sitting time; US, United States; UK, United Kingdom; WI, Wisconsin; y, years. 

**Notes:** Bold type indicates a randomized controlled trial; A/B indicates multiple intervention groups in a study. 

^a^ Published as an RCT, but not categorized as an RCT for the purposes of the current review. 

***Results of individual studies***

**Changes in movement behaviour**

Sedentary time was measured using devices in the majority (n=12) of the studies; two studies reported sitting time using self-report only.^25^,^33^ Burke et al. and Matei et al. both used the International Physical Activity Questionnaire; Matei et al. also included a second measure of sitting time using the Measure of Older Adults’ Sitting Time. Both tools have established reliability and validity for use among older adults.^35^,^36^ Changes in movement behaviours presented below were based on statistical results reported in the studies. Those that indicated changes were considered successful. 

As shown in Table 2, of the 12 groups that measured and reported within-group results for the changes in sedentary time, six groups demonstrated significant within-group changes, and six groups demonstrated no significant change; five groups did not conduct within-group statistical testing after the intervention. Within-group analyses demonstrated that physical activity improved in six studies.

**Table 2:** Changes in movement behaviours for each intervention group (within-group, pre to post)

Study	Changes in ST	Changes in PA
Aunger 2020	X	X
Blair 2021 (A)	X	X
Blair 2021 (B)	X	Steps: 1675.0/day MPA: 15.2 min/day MPA guideline bouts: 16.7 min/day^a ^
Burke 2013	ST: −50.7 min/day^a ^	Walking: 7.9%^a ^MPA: 11.9% VPA: 8.0%^a ^Strength exercises: 20.5%^a ^
Kleinke 2021	X	X
Rosenberg 2020	NA^a ^	NA
Barone Gibbs 2017	X	X
Compernolle 2020	X	X
Fitzsimons 2013	ST: −24 min/day	Stepping: 13 min/day
Gardiner 2011	ST: −3.2% STS/day: 4	LPA: 2.2% MVPA: 1%
Koltyn 2019: A	NA	NA
Koltyn 2019: B	NA	NA
Lewis 2016	ST: −51.5 min/day ST (%): −5.3% Sitting ≥ 30 min: −53.9 min/day # of bouts ≥ 30 mins: −0.8	X
Matei 2015	ST (IPAQ): −150.8 min/day ST (MOST): −143.4 min/day	Walking: 20.6 min/day
Rosenberg 2015	ST: −27 min/day ST (%): −3%	LPA (% of day): 3% MVPA: 3.7 min/day
Tosi 2021: A	NA	NA
Tosi 2021: B	NA	NA

**Abbreviations: **IPAQ, International Physical Activity Questionnaire; LPA, light-intensity physical activity; min, minutes; MOST, Measure of Older Adults’ Sedentary Time; MPA, moderateintensity physical activity; MVPA, moderate-vigorous physical activity; NA, [within-group analysis] not available; PA, physical activity; ST, sedentary/sitting time; STS, sit-to-stand transitions [or break in ST]; VPA, vigorous-intensity physical activity. 

**Notes:** X indicates no change(s) from pre to post. A/B indicates multiple intervention groups in a study. Data provided only when changes from pre to post were statistically significant from the original study. 

^a^ The intervention group reported changes that were significantly different from the change in controls (RCTs only; bolded in first column). 

For between-group results of RCTs (data not shown), only two of the five RCTs showed changes in sedentary time measures in intervention versus control groups.^25^,^27^ Burke et al. showed through regression analysis that the intervention group significantly decreased their sitting time versus controls (coefficient: −0.215, CI: −0.312 to −0.117; *p*<0.001), while Rosenberg et al. showed a difference in mean change of −58 min/day (95% CI: −100.3 to −15.6; *p*=0.007) in the intervention group versus controls. Physical activity only improved in two studies.^21^,^25^ Blair et al. reported that their intervention group B improved moderate-vigorous physical activity guideline bouts compared to controls (16.6 min/15 hours awake, 95% CI: 4.1 to 29; *p*<0.05). Compared to controls, Burke et al. reported via their regression analysis that the intervention group significantly increased participation in several physical activity outcomes: strength exercise (coefficient: 1.075, 95% CI: 0.559 to 1.591; *p* < .001), walking (coefficient: 0.909, 95% CI: 0.094 to 1.724; *p*=0.029), and vigorous-intensity physical activity (coefficient: 0.664, 95% CI: 0.128 to 1.199; *p*=0.015). 

**Intervention engagement**

The number of participants that completed the intervention was generally high; only three studies had completion rates below 80%.^21^,^25^,^34^ As shown in Table 3, ratings of satisfaction, study adherence and future commitment were also generally high. The term “future commitment” was used to describe positive answers to continuing with any part of the intervention post-study, or in recommending the intervention to others. 

**Table 3:** Intervention satisfaction, adherence and future commitment

Study	Completers (%)	Satisfaction/acceptability	Adherence/future commitment
Aunger 2020	87.5	Self-reported satisfaction (5=very satisfied): 4.5/5 (90%)	Goal adherence: 88% completion Environmental modification adherence: 52% Achieved/exceeded step targets: 42% Completed intervention: 22/24 (92%)
Blair 2021 (A)	67.0	Acceptability: 93% (27/29) “agreed”/“strongly agreed” technology increased awareness of time sitting; 79% (23/29) “agreed”/”strongly agreed” that technology (monitor + app) was easy to use; 83% (24/29) “agreed”/“strongly agreed” that technology motivated them to decrease ST	Indicated they would use the technology (monitor + app) in the future: 79% “agreed”/“strongly agreed” Wore the Jawbone monitor: 100% “very often” Checked app daily for steps: 79% “very often” Checked app for longest sedentary bout: 24% “very often”/“often” Ignored vibration to stand up: 21% “very often,” 62% “sometimes” Completed all 5 calls: 93%
Blair 2021 (B)	94.0
Burke 2013	71.0	Booklet encouraged them to think about PA: 78%	Used the exercise chart: 74% Used the exercise chart to practise the recommended exercises: 62% Calendar reminded them to consider PA: 66% Used the pedometer: 90% Used the resistance band: 63%
Kleinke 2021	83.0	NA	NA
Rosenberg 2020	100.0	Satisfied/very satisfied: 92%	NA
Barone Gibbs 2017	100.0	Reported benefiting from the program: 100%	Would definitely continue behaviour change: 74% Intended to use the armband and interface everyday: 61% Reported wearing the armband every day: 84%
Compernolle 2020	87.0	Positive feelings (being motivated, surprised and interested): 89% Intervention was not interesting or helpful: 11%	Participant reporting of daily app access: 57% System reporting of daily usage: 29% App was accessed more at the start of the intervention (3−4x, weeks 1– 2) vs. the end (1–1.5x, weeks 20–21).
Fitzsimons 2013	100.0	NA	NA
Gardiner 2011	100.0	Rated the program 8 or higher (10=extremely satisfied): 97%	NA
Lewis 2016	90.0	Overall program satisfaction: 82%	Would recommend program: 82% Achieved all of their goals: 81%
Matei 2015	85.0	NA	Returned at least 8 “tick sheets”: 92% Adherence to tips: 58%
Rosenberg 2015	69.4	Completers who reported being “somewhat”/“very satisfied” with intervention: 100%	NA
Tosi 2021 (A)	81.0	82% showed more than 70% adherence to the program	NA
Tosi 2021 (B)	82.0	NA	NA

Abbreviations: NA, not available; PA, physical activity; ST, sitting/sedentary time. 

Notes: A/B indicates multiple intervention groups in a study; bold typeface indicates a randomized controlled trial. 

**Intervention components**

Most (11/14) studies identified at least one behaviour change theory that informed intervention design. These included the social cognitive theory,^21^,^25^,^27^,^31^,^34^ self-determination theory,^24^,^32^ habit formation theory,^33^ ecological theory,^27^,^30^ behavioural choice theory^31^ and self-regulation theory.^22^ Although we aimed to extract data on the specific behaviour change techniques used in studies, we could not accurately do so, since labels varied across studies, authors reported the main techniques used only (without labelling specific intervention components) or techniques were simply not mentioned or not described. 

**Figure 2:**
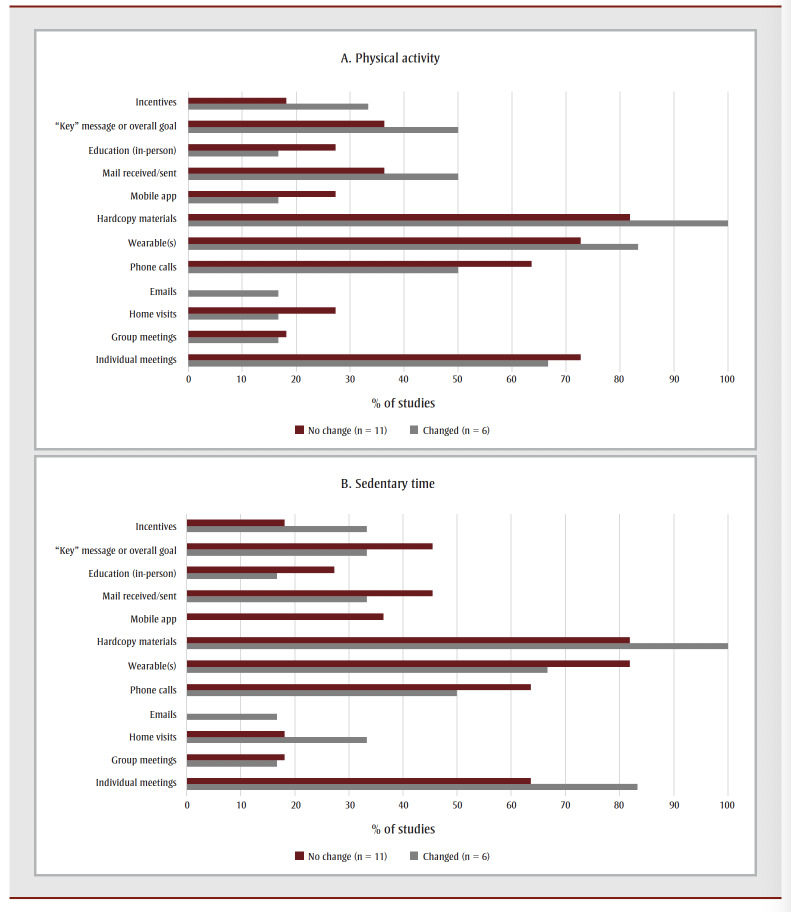
Percentage of studies in which the intervention components were used

Given the limitations with behaviour change techniques, we chose to focus on the intervention components. Table 4 summarizes the various intervention components used across studies. Intervention components used in “successful” and “unsuccessful” interventions were tallied. Figure 2 indicates the percentage of studies in which the various intervention components were used. The number of interventions that used a specific component was divided by the total number of successful or unsuccessful interventions. For example, individual meetings were used for sedentary time in seven of the nine groups that demonstrated changes in sedentary time. 

**Table 4:** Intervention components used in included studies

Study	Individual	Group	Home	Emails	Phone	Wearable(s)	Hardcopy	Mobile	Mail	Education	Key	Incentives
Aunger 2020	✓		✓		✓	✓	✓					
Blair 2021 (A)					✓	✓	✓	✓	✓		✓	
Blair 2021 (B)					✓	✓	✓	✓	✓		✓	
Burke 2013		✓		✓	✓	✓	✓				✓	✓
Kleinke 2021	✓					✓	✓		✓			
Rosenberg 2020	✓				✓	✓	✓		✓			
Barone Gibbs 2017	✓				✓	✓		✓			✓	
Compernolle 2020	✓		✓		✓	✓	✓	✓	✓	✓		
Fitzsimons 2013	✓					✓	✓					
Gardiner 2011	✓		✓			✓	✓		✓	✓	✓	
Koltyn 2019 (A)		✓				✓	✓			✓	✓	✓
Koltyn 2019 (B)		✓				✓	✓			✓	✓	✓
Lewis 2016	✓		✓		✓		✓					
Matei 2015	✓						✓					
Rosenberg 2015	✓				✓	✓	✓		✓			✓
Tosi 2021 (A)	✓				✓		✓					
Tosi 2021 (B)	✓											

Notes: A/B indicates multiple intervention groups in a study. “Individual meetings” were face-to-face, one-on-one consultations between participant and administrator. “Group meetings” were in a workshop setting facilitated by an administrator. “Home visits” were visits attended by an administrator at the participant’s home for testing/intervention purposes. “Emails” and “phone calls” indicate type of communication used for screening/contact/intervention purposes. “Wearable(s)” are any device worn by the participant for testing/intervention purposes. “Hardcopy materials” refers to any hardcopy materials used for testing/intervention purposes. “Mobile app” refers to an application on the participant’s mobile device used for testing/intervention purposes. “Mail received/sent” refers to any use of postal mail for sending/receiving materials to/from participants. “Education (in person)” refers to the use of any educational material delivered to participants during in-person consultations. “Key message/overall goal” refers to brief and simple messages directed to participants, reflecting overall intervention aims. “Incentives” were monetary compensation for participants. Bold type indicates a randomized controlled trial. 

## Discussion

Our goal was to inform the co-creation of a new intervention based on the Staircase Approach.^4^ We found that several feasible and acceptable interventions aimed at supporting community-dwelling older adults in reducing sedentary time do exist. However, these interventions have demonstrated limited success in impacting movement behaviours. Of the higher quality studies (RCTs), only two out of five demonstrated a change in movement behaviours when comparing between groups; of the lower quality studies, only 50% indicated a change in sedentary time and physical activity. This review has provided several critical insights that can inform the development of a new intervention targeting inactive, community-dwelling older adults. 

Most of the studies in our review used a behaviour change theory to guide the design of the intervention; the social cognitive theory was most commonly reported. Although there is no consensus on which behaviour change theory is best for reducing sitting time, given our understanding of environmental influences on sedentary time and physical activity,^37^,^38^ it was surprising that no studies used the social-ecological model to underpin their intervention. In a review by Heath et al.,^39^ who aimed to understand the lessons learned from evidence-based physical activity interventions, it was noted that policy and environmental approaches are critical to intervention design. Thus, it may be important to consider the use of a more comprehensive framework to design successful interventions. For example, the Stand When You Can intervention designed for older adults in assisted living settings was based on this model that incorporated environmental cues in the residence, included staff in creating a culture of movement and used individual behaviour change strategies.^40^ For older adults living in the community, the environment varies greatly, and has been shown to have a large influence on movement behaviours across different cultures and genders.^41^

Another observation that emerged during data extraction was that many studies included several behaviour change techniques in their interventions; however, these were not always clearly labelled or identified, which made analyzing these specific techniques across studies challenging. It would be helpful for researchers to describe their behaviour change interventions with reference to techniques using a universal language, for example, Michie’s behaviour change techniques taxonomy,^42^ to enable easier comparison and analysis of different interventions so that future synthesis can appropriately capture their impact.

While several interventions were considered feasible and acceptable by participants, few led to significant changes in sedentary time. Interestingly, several interventions led to changes in physical activity levels despite the focus of behaviour change efforts being on sedentary time. The goal of most interventions was to reduce total sedentary time, with some also emphasizing interrupting sitting time or standing more. This is an interesting finding in light of a previous meta-analysis that found that interventions targeting sedentary behaviours led to more meaningful changes in sedentary time than interventions that included physical activity components.^43^ It is clear that the interplay between movement behaviours is complex, and must be carefully considered in any intervention design. The unique needs of an older, community-dwelling population must also be considered. For example, a large proportion of older adults have complex chronic conditions, making it challenging to engage in physical activities of certain modes and intensities. Thus, the creation of interventions targeting interruptions in sedentary time may be critical for Step 1 of the Staircase Approach.^44^

The acceptability, satisfaction, adherence and future commitment data demonstrate that sedentary behaviour interventions have high adherence and are viewed positively by older adults. In some studies, specific intervention components were examined and showed that technology and wearables were generally well accepted in this population. This is in line with previous research that shows increasing uptake and acceptability of technology that tracks physical activity among older adults, including virtual reality–based approaches.^45^

Additionally, it is also worthy of note that our preliminary analysis of intervention components from studies in which sedentary time and physical activity changed indicated that the use of wearable technologies and workbooks may be important. Individual meetings, emails and phone calls, while resource intensive and not always feasible for larger-scale implementation, may be important to include with this population as well. 

Finally, providing key messages about goals may increase the success of interventions. Some key messages in the interventions reviewed were: “Sit less, stand more and move more, throughout the day, every day;”^21^ “Stand up and move after 30 minutes of uninterrupted sitting;”^31^ and “[B]reak up prolonged sitting (of 1 hour or more) by standing up 3 to 5times per day and progressing to 10 to 12 times per day by the 4th week.”^22^ Inclusion of these simple messages may support meaningful changes in the early stages of behaviour change. 

***Strengths and limitations***

One of the strengths of this review is its rigorous methodology, including the use of a librarian scientist and several databases. We also found a larger number of studies for inclusion in our review than anticipated, providing a robust dataset. 

However, the findings of this literature review should also be considered in light of several important limitations. First, we did not look at sex or gender differences in our analysis. This is in part because the large majority of studies did not report results by sex or gender. Given the known differences in movement behaviour patterns and preferences in men and women,^7^,^46^,^47^ this is an important future consideration for intervention design. Related to this, it is also important to note that ethnic diversity was not clearly discussed in the studies. Thus, future research is also needed to understand the impact of country and culture on the design, feasibility and uptake of such interventions. 

Second, we were unable to analyze behaviour change techniques due to inconsistencies in reporting. While many researchers clearly indicated their primary behaviour change techniques, in some instances it was impossible to determine what additional techniques were used through various intervention components (e.g. workbooks). Future research is needed to better understand the most effective behaviour change techniques when working with older adults. 

Third, it should also be noted that while we aimed to be comprehensive in our search of the published literature, we did not include a grey literature search. It is possible that we missed relevant studies as a result.

## Conclusion

We found that past sedentary behaviour interventions aimed at reducing sedentary time in community-dwelling older adults were well accepted, but inconsistent in leading to behaviour change. Nevertheless, this systematic search and review provides several important insights that can be used in the design of a new evidence-informed intervention based on the Staircase Approach. A co-creation approach is needed to ensure that the intervention design process also considers the end users, to help with adherence, feasibility, and future scalability and implementation. Future recommendations for researchers reporting on sedentary behaviour interventions include using a universal behaviour change technique taxonomy and separating analyses by age, sex and gender to investigate potential differences in males and females. 

## Acknowledgements

This work was supported by The Canadian Institutes of Health Research [grant number 17096].

We would like to thank Rogih Riad Andrawes, Roubir Riad Andrawes and Nicholas Udell for their involvement in the study. 

## Conflicts of interest

The authors have no conflicts of interest to declare.

## Authors’ contributions and statement

KK: methodology, formal analysis, investigation, data curation, writing—original draft, writing—review and editing, visualization.

MT: conceptualization, methodology, software, writing—original draft, writing—review and editing.

SH: conceptualization, methodology, writing—review and editing. 

SS: conceptualization, methodology, writing—review and editing.

BK: conceptualization, methodology, writing—review and editing. 

DD: conceptualization, methodology, writing—review and editing. 

DB: conceptualization, methodology, writing—review and editing. 

JC: conceptualization, methodology, writing—review and editing. 

SD: conceptualization, methodology, formal analysis, investigation, resources, data curation, writing—original draft, writing—review and editing, visualization, supervision, project administration, funding acquisition.

The content and views expressed in this article are those of the authors and do not necessarily reflect those of the Government of Canada. 
